# OSM/OSMR and Interleukin 6 Family Cytokines in Physiological and Pathological Condition

**DOI:** 10.3390/ijms231911096

**Published:** 2022-09-21

**Authors:** Francesca Lantieri, Tiziana Bachetti

**Affiliations:** 1 Health Science Department (DISSAL), University of Genoa, Via Pastore 1, 16132 Genova, Italy; 2 IRCCS Ospedale Policlinico San Martino, U.O. Proteomica e Spettrometria di Massa, Largo R. Benzi, 10, 16132 Genova, Italy

Oncostatin M (OSM) is a member of the interleukin-6 (IL-6) family of cytokines and can bind two different receptors, Leukemia inhibitory factor receptor (LIFR) and Oncostatin M receptor (OSMR), through a complex containing the common glycoprotein 130 (gp130) subunit.

OSMR binding recruits Janus kinases JAK1 and JAK2, leading to the activation of signal transducers and the activation of transcription factors known as STATs through multiple docking sites. The Src homologue 2 (SH2) domain of SHC Adaptor Protein 1 (Shc1) (also called p66shc), which is unique to OSMR, is also phosphorylated, inducing mitogen-activated protein kinase (MAPK) cascades (extracellular signal-related kinase ERK, p38, and c-Jun N-terminal kinase JNK). Besides the JAK/STAT and the MAPK/ERK pathways, the phosphatidylinositol-3-kinase/AKT (PI3K/AKT) and the protein kinase C delta (PKCδ) pathways are also activated [1].

OSM plays a role in several physiological and pathological conditions, including hematopoiesis, mesenchymal stem cell differentiation, fibrosis, nociception, inflammation, metabolism, and cancer [1,2,3,4,5].

In this Special Issue, several physiological and pathological conditions that implicate the IL-6 family and OSM signaling in particular have been investigated and reported in six manuscripts, all of which are briefly described and discussed below.

Bailey et al., investigated the specific effect of OSM in adipocytes since OSM is involved in human and murine obesity [6]. OSM signaling in adipocytes induces the expression of pro-inflammatory mediators, and OsmrFKO mice with adipocyte-specific *osmr* deletion are insulin-resistant and have increased adipose tissue inflammation. The authors demonstrated that OSM stimulates lipolysis in adipocytes via the p66Shc-MEK/ERK pathway, which is OSMR-mediated. The authors also showed that OSM prevents the lipolysis suppression exerted by insulin through Insulin Receptor Substrate 1 (IRS1) inhibition, which is p66Shc-ERK mediated. Accordingly, OSM stimulation in adipocyte cultures and *osmr* deletion in the adipocytes of OsmrFKO mice had opposing effects on the expression of several key lipolysis genes.

Lorchner et al., investigated OSM in myocardial infarction (MI) because OSM and LIF have been shown to have a cardioprotective role in rodent models of MI [7]. OSM activates both OSMR and LIFR in humans, while mice osm (mOSM) is generally capable of osmr binding only. Thus, the authors engineered a human-like OSM (hlOSM) protein, which signals via both OSMR and LIFR, to compare the selective effect of LIFR and OSMR in a setting that can be better translated to human clinics. Using transcriptome analysis and gene set enrichment analysis (GSEA), they showed that stimulation with hlOSM in cardiomyocyte recapitulates the effect of both mOSM and mLIF, with STAT3, STAT5, and c–Myc being the major common signaling molecules involved. HlOSM was also the most effective in STAT3 activation and in cultured cardiomyocyte survival under hypoxic conditions. The authors also showed that OSM and LIF expression increased within the infarcted heart zone in mice during the early inflammatory phase. Significant and increased expression of OSMR expression at injury sites was evident, while LIFR gradually declined within the infarction zone during the early inflammatory phase. Repetitive injections after the MI onset of mLIF, mOSM, and especially hlOSM to infarcted mice hearts prevented cardiomyocyte death, with STAT3 being more prominently activated within the infarcted zone, and STAT5 being more prominently activated in the non-injured zone. Magnetic resonance imaging (MRI) post-MI suggested a more preserved cardiac architecture and contractile function of the left ventricles of mice receiving hlOSM, not only in the injured section, but also in the non-injured regions of infarcted mice.

Jakob et al., reported that there is evidence for mOSM signaling involving both OSMR and LIFR in bone formation and resorption [8]. They showed that mOSM had a proliferative effect on a fibroblast cell line and an antiproliferative effect in bone marrow stromal cells. The effect of OSM on proliferation was mainly mediated by OSMR, with LIFR having marginal but synergistic effects on proliferation. Similar downstream pathways, such as PI3K/AKT, MAPK/ERK, and especially JAK/STAT, were activated in the two cell types. However, based on transcriptome analysis, upon mOSM stimulation, Stat1 mRNA levels were increased in bone marrow stroma cells, while Stat3 and Stat5 levels were increased in fibroblasts cells, in accordance with the proliferative effect of STAT3 and STAT5 and the antiproliferative effect of STAT1 reported in the literature. A similar enrichment in gene sets related to inflammatory phenotypes was found in both cell lines. However, in accordance with the proliferative and antiproliferative response to mOSM, the bone marrow stromal cell lines specifically showed positive enrichment in the pathways related to apoptosis and negative enrichment in the “response to growth factors” and other developmental processes. The fibroblast cells showed positive enrichment in “pyrimidine metabolism” and negative enrichment in various differentiation processes. Thus, mOSM stimulated similar pathways in the two cell lines, but the cellular response might depend on the strength and balance of the downstream OSM cytokines and their interaction with co-activators.

Bachetti et al., investigated Hirschsprung disease (HSCR)-associated enterocolitis (HAEC), a common and severe complication resulting in congenital gut malformation in HSCR [9]. HAEC is characterized by intestinal inflammation that resembles inflammatory bowel diseases (IBDs). Whole-exome sequencing (WES) on a cohort of patients affected with HAEC and compared to HSCR patients without enterocolitis revealed *OSMR*, which has already been reported to be implicated in IBDs, as the major gene affecting HAEC susceptibility, with a variant located in the OSM binding site being identified. Proteomic analysis in lymphoblasts from wild-type (wt) patients or in patients carrying this predisposing *OSMR* variant in homozygosis revealed several proteins that, following OSM treatment, were differently expressed in the variant but not in the wt sample. GSEA analysis showed that for the wt sample, a significant enrichment of the immune response pathways, including ERK1/ERK2 cascades, autophagy, and response to wounding, were completely lost in the cluster of proteins that were overrepresented in the variant sample. Other pathways enriched after OSM stimulation were related to apoptosis, catabolic processes, and DNA repair and replication. For the *OSMR* variant carrier, enrichment in several different pathways was observed, including in pathways related to lipid metabolism, tumor necrosis factor-mediated signaling pathway (TNF), JunN-terminal Kinase (JNK) cascade, the positive regulation of stress-activated Mitogen-Activated Protein Kinase (MAPK) cascade, the response to oxidative stress, and epithelium differentiation and development. Thus, proteomic analysis and GSEA confirmed the role of the OSM–OSMR axis in HAEC susceptibility identified by WES, likely through an impaired immune system response and an unbalance of the gut homeostasis, which has already been suggested to underlie the acute inflammation in HAEC.

De Souza et al., reviewed the role of OSM in bone mass regulation and in bone resorption in particular and also discussed how the different signaling processes mediated by OSM through the two receptors OSMR and LIFR can play different roles in osteoclastogenesis [10]. In vitro studies suggest that mOSM stimulates osteoclast formation through OSMR binding. However, in vivo experiments are more conflicting, indicating OSM as a stimulator of both bone resorption and bone formation. The authors summarize that the OSM–OSMR binding increases Receptor Activator of Nuclear Factor κ B ligand (RANKL) expression and osteoclast formation through the Shc1/STAT3/ERK pathway. OSM–LIFR binding would instead act through the Src homology region 2 domain containing phosphatase-2 (SHP2), for which OSMR lacks the recruitment motif. The SHP-2/JNK-activated pathway might suppress sclerostin expression in osteocytes and can thus be an osteoclast inhibitory pathway and increase bone formation. The authors recently demonstrated that OSM induces the expression of Wnt Family Member 16 (WNT16) through the OSMR/gp130/Shc1/STAT3 pathway, and WNT16 has an inhibitory effect on RANKL-induced osteoclast differentiation. Thus, they propose that OSM could be an osteoclastogenesis activator but that an excess of OSM would induce the expression of WNT16, which would shift the balance between OSM’s catabolic response of bone resorption and anabolic response of bone formation.

Nummenmaa et al., mainly focused their work on the finding that the upregulation of IL-6 expression in osteoarthritis (OA) is mediated by Transient receptor potential ankyrin 1 (TRPA1); they obtain results through RNAseq analysis by comparing chondrocytes from wild-type TRPA1 mice and TRPA1 knockout mice [11]. The observations achieved by the omics approach were further supported and validated through several additional experiments. Thus, the authors suggest TRPA1 as a drug target in osteoarthritis.

Most of the articles highlighted in this editorial highlight the importance of OSM in the balance of signaling to maintain tissue health and its dual effect in many physiological processes. In particular, Bailey et al., suggest that intact adipocyte OSM–OSMR signaling is necessary for maintaining adipose tissue health. Bachetti et al., confirm that OSM/OSMR might be involved in maintaining gut homeostasis, the unbalance of which might lead to enterocolitis episodes in HSCR. De Souza et al., highlighted the possible role of OSM in the balance between anabolic and catabolic processes in bone metabolism. According to this specific balance effect, Jakob et al., described the opposite effect of mOSM on cell proliferation depending on the cell type. Finally, Lorchner et al., showed the cardioprotective effect of mOSM in infarcted myocytes, which is necessary both in the early inflammatory phase and a following reparative phase of myocardial healing.

The dual effect of OSM might depend on many factors: the specific binding receptor and the use of specific docking sites, the cellular environment, and the subsequent activation of downstream cytokines. All of these aspects are addressed in the manuscripts included in this Special Issue. De Souza et al., specifically describe how the different signaling processes mediated by OSM through the two receptors OSMR and LIFR can play different roles in osteoclastogenesis. This alternative cascade signaling provides a reasonable explanation for the balance between the catabolic and the anabolic bone responses. Lorchner et al., found STAT3, STAT5, and c–Myc to be the major common signaling molecules involved in cardiomiocytes, but with different behaviour in response to mOSM, mLIF, and hlOSM and a different effect in cardiomyocyte survival in vitro and at distinct activation sites within the myocardium after infarction. Since human OSM binds both OSMR and LIFR, the higher effect of hlOSM with respect to mOSM and mLIF suggest additive cardiomyocyte protection in infarction via the simultaneous activation of OSMR and LIFR in ischemic hearts during the early inflammatory phase, with an additional LIFR-mediated indirect protection loop. This complex non-redundant and environmentally dependent activity of OSM and LIF at distinct sites of the injured heart suggests receptor binding specificity and cross talk, which might also explain the dual effects of OSM. Unfortunately, no other study has investigated the distinct action of OSMR/LIFR. However, Bachetti et al., found differently enriched downstream pathways upon OSM stimulation depending on the presence of a variant that likely affects OSMR function: these differences in pathway activation confirm that OSMR mediates OSM activity differentially to activate downstream pathways. Jakob et al., show that OSM action on cellular proliferation is mediated by OSMR through known pathways, with a pro- or the anti-proliferative effect depending on the cellular environment. STAT3 and STAT5 expression and activation is modulated in different ways in fibroblasts and in bone marrow stromal cells in response to mOSM; thus, the authors propose that responses to mOSM might depend on specific balances and ratios of STAT proteins and their interaction with co-activators. To note, both STAT5 and STAT3 are critical mediators of adipocyte lipid metabolism, although Bailey did not examine the JAK-STAT pathway in the study included in this Special Issue.

Besides the different biological roles described here, OSM is known to be involved in inflammation, having both anti- and pro-inflammatory effects. Jakob et al., found enrichment in the gene sets related to an inflammatory phenotype in both fibroblasts and bone marrow upon mOSM treatment in addition to pathways specific to each cell line and in accordance with the proliferative effects detected. Similarly, Bachetti et al., found pathways related to immune response, apoptosis, response to wounding, and inflammation among those enriched upon OSM stimulation. Moreover, the biological OSM functions investigated by Lorchern et al., and by Bailey et al., are also closely connected with inflammatory processes: Lorchert et al., investigated the healing inflammatory response at the myocardium and cite how the upregulation of anti-apoptotic and anti-oxidant proteins is crucial for cardiomyocyte survival; Bailey et al., point out that OSM induces adipocyte lipolysis and promotes insulin resistance in obesity, yet the OSMR loss and the loss of OSM-induced lipolysis in adipocytes still result in adipose tissue inflammation and insulin resistance.

Interestingly, connections between several inflammatory diseases can be suggested. OSM has already been implicated in IBDs. Bachetti et al., show the involvement of the OSM–OSMR axis in HAEC, a complication associated with the intestinal genetic disease HSCR, which clinically resembles IBDs. Moreover, an increased occurrence of IBDs in HSCR patients has been reported [12]. De Souza et al., also report that OSM has an effect on osteoclast stimulation in inflammatory diseases. This observation, following the results of Bachetti et al., implies the question of whether the Reduced Bone Mineral Density (BMD) observed in IBDs, in which OSM is a worse prognosis factor, may be ascribed to the osteclastogenetic role of this cytokine. This hypothesis is in accordance with what was reported by Fernandes et al., who show that osteoarthritis (OA) and osteoporosis (OP) are the most frequently occurring non-inflammatory rheumatic conditions observed in ulcerative colitis and Crohn’s disease [13]. It is not hard to find a relationship with the paper by Nummenmaa et al., which describes the role of TRPA1 in upregulating IL-6 expression in osteoarthritis (OA). Kun et al., reported the upregulation of the TRPA1 ion channel in inflamed human and mouse colon, thus suggesting a link between the role of TRPA1 in IBDs and in OA and OP. In particular, TRPA1 upregulation and activation in colitis could exert protective effects by decreasing the expression of several proinflammatory neuropeptides, cytokines, and chemokines [14]. TRPA1 is also a negative regulator of adipogenesis, thus acting in obesity prevention. This observation further calls back to the role of OSM in lipolysis and in insulin resistance [15].

In conclusion, OSM exerts a variety of functions in several biological processes. The articles included in this Special Issue also exemplify the important functions that OSM plays in many different cells and tissues, such as in adipocytes, osteoblasts, fibroblasts, bone marrow stromal cells, and chondrocytes, and thus in bone, adipose tissue, and even in the gut. Accordingly, OSM is expressed by many cell types, including macrophages, dendritic cells, neutrophils, T cells, muscle cells osteoblasts, and osteocytes. LIFR is expressed by various cell types, including in hematopoietic cells, while OSMR expression is instead more specifically found in mesenchymal cells such as fibroblasts, endothelial cells, osteoblasts, and epithelial cells as well as in several types of cancer cells [1]. Nonetheless, Bachetti et al., showed an effect in lymphoblasts as well; thus, even low levels of OSMR might act in several types of cells.

This is particularly relevant since OSM and the OSM–OSMR axis might be drug targets in a variety of conditions. OSM has already been therapeutically targeted in rheumatoid arthritis (RA) [16] and has been suggested as a possible therapy target for squamous cell carcinomas [17] and IBDs [18]. It might also be relevant in protection from myocardium infarction by preserving the functional and structural integrity of the damaged heart, as discussed by Lorchner et al., Since OSM is a lipolysis inducer in vitro, it should be further addressed in the context of obesity and metabolic disease, as suggested by Bailey. Finally, due to the similarities between HAEC and IBDs, it deserves particular attention in HAEC as well.

The role of OSM–OSMR as therapeutic targets should take into account the possible negative effects due to the multitude and variable effects of the axis depending on the tissue and cellular context. Moreover, the wide range of effects of OSM–OSMR should also be considered to avoid side effects associated with broad OSM blockage.

All of these considerations stress the need to further investigate OSM effects such as aberrant proliferation, inflammation, and immune system modulation in various biological contexts to elucidate the complex OSM network.
